# Mining the soluble chloroplast proteome by affinity chromatography

**DOI:** 10.1002/pmic.201000495

**Published:** 2011-02-25

**Authors:** Roman G Bayer, Simon Stael, Edina Csaszar, Markus Teige

**Affiliations:** Department of Biochemistry and Cell Biology, Max F. Perutz Laboratories, University of ViennaAustria

**Keywords:** Affinity chromatography, ATP-binding, Chloroplast, Metal-binding, Plant proteomics, YFP-fusion protein

## Abstract

Chloroplasts are fundamental organelles enabling plant photoautotrophy. Besides their outstanding physiological role in fixation of atmospheric CO_2_, they harbor many important metabolic processes such as biosynthesis of amino acids, vitamins or hormones. Technical advances in MS allowed the recent identification of most chloroplast proteins. However, for a deeper understanding of chloroplast function it is important to obtain a complete list of constituents, which is so far limited by the detection of low-abundant proteins. Therefore, we developed a two-step strategy for the enrichment of low-abundant soluble chloroplast proteins from *Pisum sativum* and their subsequent identification by MS. First, chloroplast protein extracts were depleted from the most abundant protein ribulose-1,5-bisphosphate carboxylase/oxygenase by SEC or heating. Further purification was carried out by affinity chromatography, using ligands specific for ATP- or metal-binding proteins. By these means, we were able to identify a total of 448 proteins including 43 putative novel chloroplast proteins. Additionally, the chloroplast localization of 13 selected proteins was confirmed using yellow fluorescent protein fusion analyses. The selected proteins included a phosphoglycerate mutase, a cysteine protease, a putative protein kinase and an EF-hand containing substrate carrier protein, which are expected to exhibit important metabolic or regulatory functions.

## 1 Introduction

Chloroplasts are semi-autonomous organelles of endosymbiotic origin found in all plant and algal cells. They have essential roles in processes such as photosynthesis, biosynthesis of amino acids and vitamins, lipid synthesis, or storage of starch. Analysis of the chloroplast proteome helps to elucidate the multitude of chloroplast functions by providing information about the protein composition and compartmentalization of metabolic pathways 1–4.

Beginning with the completion of the genome sequence of *Arabidopsis thaliana* in the year 2000 various efforts have been made to estimate the size of the chloroplast proteome using sequenced-based prediction programs. The *Arabidopsis* Genome Initiative calculated an overall number of ∼3600 chloroplast proteins using TargetP 5, whereas usage of ChloroP resulted in the prediction of ∼1900–2500 chloroplast proteins 6. This difference can be explained by the fact that chloroplast transit peptides (cTPs) do not share distinct consensus motifs in their primary structure and by their remarkable diversity 7. Therefore, an improved prediction strategy was applied accepting cTPs only when they were identified by at least three out of four different programs 8. This resulted in the prediction of ∼2100 proteins, which probably fits best to the actual size of the chloroplast proteome. However, as reliable information on the subcellular localization of proteins cannot be deduced from genome sequences alone 1, 4, it is indispensable to analyze the chloroplast proteome experimentally.

Since the first plant genomes were published, large-scale MS-coupled proteomic approaches have routinely been employed to directly detect proteins in organellar preparations 9, and the obtained data have been integrated into several protein databases. For example, the Plant Proteome Database (PPDB) contains ∼1200 manually curated chloroplast proteins including data of a recently published chloroplast study, which claims to be the most comprehensive chloroplast proteome analysis to date 10, 11. Thus, PPDB provides by far the most extensive, curated resource for experimentally verified chloroplast-localized proteins. In combination with protein data from a recently published chloroplast proteomic study integrated into the novel database AT_CHLORO 12, both databases make up a total of ∼1700 unique chloroplast-localized proteins. This number probably reflects the amount of chloroplast proteins that is accessible with the current MS technologies and traditional preparation techniques.

Up to date, neither the proteome of an organism nor an organelle has been experimentally identified completely. This is due to the inaccessibility of certain proteins to proteomic techniques as a consequence of their physicochemical properties and the dynamic range of proteins (10^6^ magnitudes) leading to a repeated detection of abundant proteins. To overcome the dynamic range problem, it is necessary to modify the fractionation techniques prior to MS 1. In accordance with Ferro et al. 12 we think that classical large-scale chloroplast proteomic approaches have reached their limit and only directed approaches have the potential to unveil low-abundant proteins. To date, there are only very few reports about studies aiming at the targeted identification of organellar proteins present in the literature. Examples are the identification of thioredoxin-interacting proteins in the stroma of chloroplasts by using immobilized thioredoxin affinity columns and the analysis of ATP-binding proteins in chloroplast membranes or in the mitochondrial matrix by ATP-affinity chromatography 13–16.

**Figure 1:**
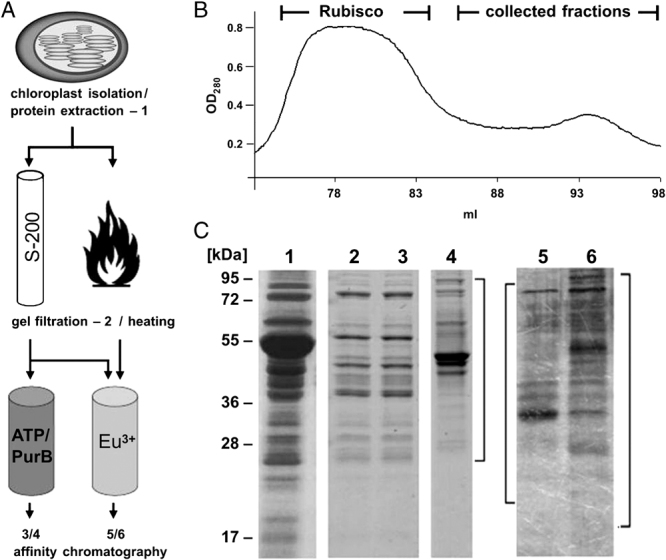
Experimental strategy and procedure. A, Flow scheme. B, Elution profile of gel filtration. *X*-axis shows milliliters of eluting sample. *Y*-axis shows absorbance at 280 nm indicating relative protein content. C, Affinity chromatography. 1–6, protein samples analyzed by SDS-PAGE: 1, crude chloroplast protein extract. 2, sample after gel filtration prior to affinity chromatography. 3, sample after heating. 4, elution of ATP-affinity column. 5, citrate elution of Eu^3+^-affinity column. 6, EDTA strip of Eu^3+^-affinity column. In lanes 4–6 the region, where protein bands were cut, is indicated.

We set out to identify novel, low-abundant soluble proteins localized in the chloroplast by applying a targeted fractionation approach prior to protein detection by MS. In order to reduce the sample complexity we decided to implement a two-step strategy (Fig. 1A). In a first step, we either performed SEC of extracted stroma proteins, or we performed a heat treatment of isolated chloroplasts. Both strategies led to an almost complete separation of the most abundant protein ribulose-1,5-bisphosphate carboxylase/oxygenase (Rubisco) from the rest of the soluble proteins. In a second step, we performed affinity chromatography using different ligands, which not only further reduced the complexity of the sample but also allowed a specific enrichment of proteins according to their biological function 17. In the end we were able to detect a subset of ∼20% of the expected 2100 chloroplast proteins including novel chloroplast-localized proteins. The chloroplast localization of 13 selected candidate proteins was confirmed by yellow fluorescent protein (YFP) fusion analysis.

## 2 Materials and methods

### 2.1 Chloroplast isolation – *A. thaliana*

A comparison of several published chloroplast isolation protocols revealed that an adapted version of the protocol by Kunst 18 resulted in the highest yield of intact chloroplasts. Briefly, *Arabidopsis* plants were grown for approximately 8 wk under short day conditions (8 h light/16 h dark photoperiod at 100–150 μmol m^−2^ s^−1^, 22±5°C, humidity 60±20%). Leaves were harvested and homogenized in the HB buffer (450 mM sorbitol, 20 mM Tricine, 10 mM Na_2_EDTA, 5 mM NaHCO_3_, 0.1% BSA, 10 mM isoascorbic acid, 1 mM reduced glutathione, pH 8.4 with KOH) using a Waring blender (3 pulses: low–low–high; 2–3 s each). In comparison to the original chloroplast isolation protocol, addition of isoascorbic acid and glutathione to the homogenization buffer resulted in a significant increase in the yield of intact chloroplasts. After filtration and centrifugation the chloroplasts were purified over continuous Percoll gradients, which consisted of Percoll (GE Healthcare) mixed in a 1:1 ratio with 2×RB buffer (600 mM sorbitol, 40 mM Tricine, 10 mM MgCl_2_, 5 mM Na_2_EDTA, pH 7.6 with KOH). The gradient was formed by centrifugation for 30 min at 53 000×*g* and then the chloroplasts were centrifuged for 6 min at 10700×*g*. Intact chloroplasts were recovered from the gradient, washed with 1×RB buffer and stored at −80°C.

### 2.2 Chloroplast isolation – *Pisum sativum*

The chloroplast isolation procedure was adapted from 19. Briefly, *P. sativum* plants were grown for 8–9 days under long day conditions (16/8 h photoperiod at ∼70 μmol m^−2^ s^−1^, 21±5°C, humidity 70–90%). Leaves were cut and homogenized using a Waring blender. The homogenate was filtered through Miracloth (Merck, Germany) and centrifuged. The resuspended pellets were loaded on top of 2–4 preformed Percoll step gradients consisting of 12 mL 40% Percoll and 7 mL 80% Percoll (in 330 mM sorbitol, 50 mM Hepes/KOH pH 7.6). After centrifugation intact chloroplasts were recovered from the 40–80% interphase and washed. Isolated chloroplasts were pooled and immediately frozen in liquid nitrogen and stored at −80°C.

### 2.3 Stromal protein extraction and gel filtration

Chloroplasts (∼20 mg of chlorophyll; measured according to Arnon 20) were incubated in the breaking buffer (10 mM Tricine pH 8, 10 mM MgCl_2_, 1 mM DTT, supplemented with protease inhibitor cocktail Complete Mini EDTA-free (Roche Applied Science) on ice for 5 min. After centrifugation for 6 min (12 000×*g*, 4°C) the supernatant was transferred to a new tube and the extraction was repeated. Subsequently, the extracts were pooled and the buffer was exchanged to buffer A (50 mM Tris pH 7.8, 50 mM NaCl, 10 mM MgCl_2_) using PD-10 Desalting columns (GE Healthcare). The sample was concentrated to ∼500 μL using a Centriprep Centrifugal Filter Unit (NMWL: 10 kDa; Millipore). After clarification by centrifugation for 10 min (16 100×*g*, 4°C) the supernatant was applied to a Superdex 200 (GE Healthcare) gel filtration column and SEC was performed on an FPLC system (GE Healthcare) at a flow rate of 0.8 ml/min (buffer A).

### 2.4 Heat treatment of isolated chloroplasts and protein extraction

Isolated pea chloroplasts were lyzed by addition of 7 mL of lysis buffer (20 mM DTT, 0.1% Triton X-100, protease inhibitor cocktail Complete Mini EDTA-free) to 3 mL of chloroplasts (containing ∼4 mg/mL chlorophyll) and incubation for 10 min on ice. The chloroplast suspension was divided into 1 mL aliquots, rapidly heated to 75°C for 5 min, and immediately cooled on ice. Heat-denatured proteins and thylakoid membranes were pelleted by centrifugation at 20 000×*g* for 10 min. After centrifugation for 30 min at 100 000×*g* (TLS55 rotor, Optima Ultracentrifuge; Beckman Coulter) and 4°C, the supernatant was rebuffered to IDA column-loading buffer (100 mM Tris-HCl pH 7.5, 3 M NaCl, 200 mM CaCl_2_) on a PD-10 column (GE Healthcare).

### 2.5 Affinity chromatography ATP/Purvalanol B (Pur B)

C10-linked Aminophenyl-ATP-Sepharose was purchased from JENA Bioscience (Jena, Germany). Preparation of PurB affinity sepharose was done as previously described 21. In both cases the affinity sepharose was poured into disposable polystyrene columns (Thermo Scientific) and the columns were run by gravity flow at room temperature.

*PurB column*: The column (500 μL of slurry) was equilibrated with 10 column volumes of PurB buffer (buffer A + 350 mM NaCl, 0.5% Triton X-100). Nearly, 1.5 mg protein sample (gel-filtrated chloroplast stroma) was adjusted by the PurB buffer and then applied to the column. Subsequently, the column was washed with 20 column volumes of the PurB buffer and bound proteins were eluted with 6 column volumes of 0.5% SDS.

*ATP column*: The column (500 μL of slurry) was equilibrated with 10 column volumes of ATP buffer (buffer A + 100 mM NaCl, 0.05% NP-40). Nearly, 1.5 mg protein sample (gel-filtrated chloroplast stroma) was adjusted by the ATP buffer and then applied to the column. Subsequently, the column was washed with 20 column volumes of the ATP buffer and bound proteins were eluted with 6 column volumes 0.5% SDS.

All fractions were precipitated with TCA using a standard protocol (LabFAQS, http://www.roche-applied-science.com/labfaqs/intro.htm). The pellets were resuspended in the SDS loading buffer and the precipitated proteins were resolved by a 12% SDS-PAGE. Proteins were visualized by Coomassie or silver staining (using formaldehyde instead of glutaraldehyde), and bands were excised and subjected to MS.

### 2.6 Eu^3+^-IDA column affinity chromatography

The Eu^3+^-IDA column affinity chromatography was adapted from 22. To prepare the Eu^3+^-IDA affinity column, a disposable polystyrene column was filled with 1 mL of IDA-sepharose (Thermo Scientific) and washed with 5 mL of 100 mM EDTA (pH 7.0), followed by 10 mL of double distilled water. A 50 mM EuCl_3_ (Alfa Aesar, USA) solution was applied to the column, followed by washing with 25 mL double-distilled water. The column was equilibrated with 10 mL of the equilibration buffer (100 mM Tris-HCl pH 7.5, 2 M NaCl, 200 mM CaCl_2_).

Samples were obtained either from SEC of chloroplast stroma or from heat denaturation of isolated chloroplasts. In the case of fractionated stroma salts were added to match the IDA column-loading buffer. After loading of a sample, the column was washed with 10 mL equilibration buffer, 5 mL of sulfate buffer (600 mM Na_2_SO_4_, 100 mM Tris-HCl pH 7.5, 2 M NaCl) and 2.5 mL of malonate buffer (40 mM malonate, 600 mM Na_2_SO_4_, 100 mM Tris-HCl pH 7.5, 2 M NaCl). Protein was eluted with a citrate solution (0.2 M phosphate buffer pH 7.5, 3 M NaCl, 200 mM citrate) and afterwards the column was stripped with 100 mM EDTA.

The citrate eluate and EDTA-strip were buffer-exchanged to 50 mM Tris-HCl pH 7.5 on a PD-10 column and precipitated in four volumes of cold acetone. Eluted proteins were separated by SDS-PAGE and visualized by silver staining. Bands were excised and subjected to MS.

### 2.7 MS analyses

Coomassie or silver-stained gel bands were used for the nano-electrospray LC-MS/MS analyses as described previously 23. The gel bands were cut out, and in case of Coomassie stained bands destained with a mixture of ACN and 50 mM ammonium hydrogen carbonate. Proteins were reduced by DTT and alkylated by iodoacetamide. Trypsin was used as protease. Samples were digested overnight at 37°C and the digest was stopped by addition of 10% formic acid in water to an end concentration of approximately 1%.

Peptides were separated on an UltiMate™ HPLC system (Dionex) equipped with a PepMap C18 column (300 μm×5 mm) and a 75 μm×150 mm analytical column of the same material. About 0.1% TFA was used for binding of the peptides and elution was performed using a linear gradient of ACN and 0.1% formic acid in water. LC-MS/MS analyses were carried out using the UltiMate™ system interfaced to an LTQ (Thermo Scientific) linear ion trap mass spectrometer. The electrospray voltage was set to 1500 V and peptide spectra were recorded over the mass range of *m/z* 450–1600. MS/MS spectra were recorded in information-dependent data acquisition with a default charge state set to 3. The mass range for MS/MS measurements was calculated according to the masses of the parent ions. One full spectrum was recorded followed by four MS/MS spectra for the most intense ions, automatic gain control was applied and the collision energy was set to the arbitrary value of 35. Helium was used as the collision gas. Fragmented ions were set onto an exclusion list for 20 s. Raw spectra were interpreted by Mascot 2.2.04 (Matrix Science) using Mascot Daemon 2.2.2. The peptide tolerance was set to ±2 Da, MS/MS tolerance was set to ±0.8 Da. Carbamidomethylcysteine was set as static modification, oxidation of methionine residues was set as the variable modification. Trypsin was selected as protease and two missed cleavages were allowed.

MASCOT results were loaded into Scaffold (Ver. 2.01.01.1; Proteome Software) for an X! Tandem Search. Peptide identifications were accepted, if they could be established at greater than 95% probability as specified by the Peptide Prophet algorithm 24. Protein identifications were accepted, if they could be established at greater than 99% probability as assigned by the Protein Prophet algorithm 25. Additionally, at least two identified peptides were required. Proteins were identified from the full genome sequence of TAIR in the case of *Arabidopsis* samples and from the recently created EST database in the case of pea samples 26.

### 2.8 Data validation

Identified proteins (always referring to *Arabidopsis* gene identifier (AGI) codes) were imported into Microsoft Excel for further analyses. Redundant protein identifications were removed using the advanced filter. Proteins were searched against PPDB 10, plprot 27, AMPDB 28, SUBA 29 and AraPerox 30 databases. All proteins not found in any of the abovementioned databases were manually inspected regarding experimental verification of subcellular localization by searching in publications found in the TAIR AGI entry (http://www.arabidopsis.org) or in the ENTREZ search engine (http://www.ncbi.nlm.nih.gov/sites/gquery). Targeting prediction was done with TargetP 31, ChloroP 32, Aramemnon consensus prediction 33 and MultiP 34.

To test for the presence of a P-LOOP motif in proteins, a regular expression of the motif, which was obtained from the PROSITE database 35, was created using Microsoft Excel and queried against all protein sequences (TAIR8 release). Furthermore, the nucleotide- and metal-binding features of identified proteins were individually analyzed using the annotated protein function and the databases PROSITE and ENZYME 35, 36.

### 2.9 Subcellular localization studies

The coding sequences of the analyzed candidate genes were obtained by RT-PCR from total leaf RNA or in the case of OTL by PCR from a RIKEN BRC *Arabidopsis* Full-Length clone (RAFL21-73-A21) 37–39. C-terminal YFP-fusions of the candidate genes were cloned into the binary plant expression vector pBIN19 40. Tobacco transfection and subcellular localization analysis were done as previously described 41.

## 3 Results and discussion

### 3.1 Enrichment and identification of low-abundant chloroplast proteins

As most chloroplast proteomics studies focussed on the exploration of the thylakoid protein complement, mining the soluble proteome has the highest potential to discover new proteins. Furthermore, soluble proteins are easily accessible by standard chromatographic separation techniques in contrast to hydrophobic proteins originating from thylakoid preparations.

We decided to use chloroplasts isolated from pea, because they are known to be highly pure and intact in contrast to *Arabidopsis* chloroplasts, which tend to break and lose their stromal content during isolation 42. As the pea genome has not been sequenced yet, we employed a recently created pea EST database that already proved to be useful in proteomic studies of the chloroplast envelope 26.

In a first step, after extraction of stromal proteins from isolated pea chloroplasts, we performed gel filtration in order to enrich for low-abundant proteins. By this means, we separated the most abundant protein, the multimeric Rubisco protein complex with a size of ∼540 kDa, from the majority of other proteins that are of much smaller size (Fig. 1B). Compared to other purification strategies, such as employing Rubisco antibody columns, SEC had the advantage that also ribosomes were removed 43. This led to a depletion of the abundant ribosomal proteins, which would normally exacerbate the detection of low-abundant proteins.

In a second purification step, we subjected the pooled fractions eluting after the prominent Rubisco peak from the gel filtration column to affinity chromatography. This method is based on the specific and reversible interaction of a ligand with its target protein, thus presenting a major advantage over the multidimensional protein identification technology MudPIT, which is applied to peptide mixtures 44.

We combined the selection of ligands with the general interest in understanding cellular signaling including protein kinases 45, and extended our approach to ATP-binding proteins as a whole. Therefore, we used ATP and the ATP-binding site directed protein kinase inhibitor PurB as ligands in independent chromatographic runs. Additionally, we used a ligand specific for metal-binding proteins. Initially, we aimed at calcium-binding proteins, but it is known that Ca^2+^ easily gets desorbed from affinity matrices in a process called metal ion transfer. Hence, we used the ligand Eu^3+^, which in contrast to Ca^2+^ was demonstrated to be stably attached to the affinity matrix, and which is even able to adsorb calcium-binding proteins 22. The Rubisco-depleted fractions after gel filtration were applied to all three different affinity ligands.

As an alternative to SEC, we performed a heat treatment of isolated chloroplasts and recovered soluble proteins and soluble fragments of membrane proteins after centrifugation. Originally, this step was established to enrich for heat-stable calmodulins 46, but empiric results in our lab showed that this procedure was also very efficient for the removal of Rubisco resulting in an enrichment particularly of heat-stable proteins. However, in contrast to SEC, heating did not lead to a depletion of ribosomes. After the heat treatment the sample was applied only to the Eu^3+^-column.

In order to achieve a maximal resolution for the subsequent protein identification by MS, eluted proteins from all three affinity columns were further separated by SDS-PAGE (Fig. 1C). A comparison of the original sample to the eluting fractions revealed a specific enrichment of proteins. Separated gel lanes of all eluting fractions were cut into slices and after extraction and digestion proteins were identified by MS/MS using the pea EST database 26. Each identified protein was queried against the *Arabidopsis* genome database and the corresponding AGI of the closest homologue was determined. All further analyses were carried out using the respective *Arabidopsis* genes.

### 3.2 Saturation of protein identifications

The analysis of three biological replicates and several technical replicates resulted in the identification of 448 unique proteins with high confidence (Supporting Information Table S1). Based on all obtained results we calculated saturation curves referring to identified proteins (Supporting Information Fig. S1). For each affinity strategy we analyzed three biological samples and plotted the percentage of all new identified proteins per sample. Using the ATP-affinity strategy we identified in total 319 proteins. Already, 82% of all proteins were identified in the first biological sample, and the second biological replicate led to the detection of only additional 4%. A significant improvement in the discovery of new proteins (14%) could be obtained only after changing the ligand from PurB to ATP for the third biological replicate.

Similar results were obtained using the Eu^3+^-column. In total, we identified 273 proteins. 54% of all proteins were discovered in the first biological sample, and the second biological sample gave again no significant improvement (only 1%). In both cases heat-treated chloroplast extracts were applied to affinity chromatography, whereas gel-filtrated stroma extracts were used for the third biological replicate. This led to a significant increase in newly identified proteins (45% of all identified proteins).

### 3.3 Subcellular localization of identified proteins

In order to get an idea about the enrichment of chloroplast-localized proteins in our data set, we analyzed the number of predicted chloroplast proteins using TargetP. Out of the 448 identified proteins 84.3% are predicted to contain a cTP compared to 14.9% proteins of the whole *Arabidopsis* proteome (TAIR9 release). Furthermore, to assess the quality of our data set regarding the amount of already experimentally verified chloroplast proteins and non-chloroplast contaminants, we queried available organellar protein databases. We used the databases PPDB and plprot 27, which focus on chloroplasts, the mitochondrial AMPDB 28, the peroxisomal AraPerox 30 and the database SUBA 29, which integrates data of all subcellular compartments. The localization of all remaining proteins, which were not found in any database, was manually curated. Only if no experimental information on the subcellular localization of a protein could be found, it was considered to be a putative novel chloroplast protein.

Overall, this analysis revealed a good quality of our chloroplast isolations as reflected by the high rate of known chloroplast proteins being 84% (376 proteins) and the low contamination rate of 6.5% (29 proteins). It is important to note here that dual targeting 47 is not considered and therefore the real contamination rate will most likely be lower. In total, 9.6% (43 proteins) were classified as putative new to the chloroplast (Table 1). Notably, knockout mutants of eight of these proteins in *Arabidopsis* do exhibit a chloroplast-related phenotype according to the Chloroplast 2010 database (http://www.plastid.msu.edu).

**Table 1:** The 43 identified putative novel chloroplast proteins

AGI code	Functional annotation (TAIR9)	TargetP	ATP/PurB	Eu^3+^	Chloroplast 2010
**AT1G06510**	**Unknown protein**	**C**	−	+	**WP**
**AT1G15730**^a^,b)	**PRL1-interacting factor L, putative**	**C**	+	−	**WP**, **CF**
**AT1G19920**a)	**ATP sulfurylase**	**C**	+	−	**–**
AT1G21500a)	Chloroplast Unknown protein 1	C	−	+	**–**
**AT1G22410**	**2-Dehydro-3-deoxyphosphoheptonate aldolase**	**C**	+	+	**WP**, **SAA**
AT1G23800	ALDH2B7; 3-chloroallyl aldehyde dehydrogenase (NAD)	M	+	−	**–**
AT1G30510a)	ATRFNR2; root FNR 2)	C	+	−	**–**
AT1G36280^a^,b)	Adenylosuccinate lyase	C	+	−	WP
AT1G42430	Chloroplast Unknown protein 1	O	+	+	**–**
AT1G54310	RNA binding	M	+	−	**–**
AT1G60000^a^,b)	29 kDa ribonucleoprotein	C	+	+	**–**
AT1G66530	Arginyl-tRNA synthetase, putative	O	+	−	**–**
AT1G71720b)	S1 RNA-binding domain-containing protein	C	−	+	**–**
AT1G71920a)	Histidinol-phosphate aminotransferase, putative	C	+	−	**–**
AT1G74920	ALDH10A8; 3-chloroallyl aldehyde dehydrogenase	O	+	−	**–**
AT1G76690	OPR2; 12-oxophytodienoate reductase	O	+	−	**–**
AT1G77122	Unknown protein	C	+	+	**–**
**AT1G77670**a)	**Aminotransferase class I and II family protein**	**M**	+	−	**–**
AT1G77930	DNAJ heat shock N-terminal domain-containing protein	C	−	+	WP
AT1G79530c)	GAPCP-1; glyceraldehyde-3-phosphate dehydrogenase	C	+	−	WP, LFA
AT1G79870a)	Oxidoreductase family protein	O	+	−	**–**
**AT2G17240**	**Unknown protein**	**C**	−	+	**–**
AT2G17340	Pantothenate kinase-related	O	+	−	**–**
AT2G21350	RNA binding	C	−	+	**–**
AT2G23390	Acyl-CoA N-acyltransferase	M	+	−	**–**
**AT2G25870**	**Haloacid dehalogenase-like family protein**	**M**	+	−	**–**
AT2G31890b)	ATRAP; putative RNA binding domain	C	+	−	**–**
AT2G44760	Unknown protein	C	+	−	**–**
AT3G02900a)	Unknown protein	C	−	+	**–**
**AT3G04650**	**FAD-dependent oxidoreductase**	**C**	+	−	**–**
AT3G25110	AtFaTA; *Arabidopsis* FatA acyl-ACP thioesterase	C	+	−	**–**
AT3G29185a)	Unknown protein	C	+	+	**–**
AT3G55870	Anthranilate synthase, α subunit, putative	S	+	−	**–**
**AT3G57810**	**OTU-like cysteine protease family protein**	**M**	−	+	**–**
AT3G59040	Pentatricopeptide (PPR) repeat-containing protein	C	+	−	**–**
AT4G27070a)	TSB2; tryptophan synthase β subunit 2	C	+	+	WP
AT5G02590	Tetratricopeptide (TPR) repeat-containing protein	C	−	+	**–**
AT5G14460	Pseudouridine synthase/transporter	C	+	−	**–**
AT5G15390	tRNA/rRNA methyltransferase (SpoU) family protein	C	+	−	**–**
**AT5G22620**^a^,b)	**Phosphoglycerate mutase family protein**	**C**	+	−	**LAA**
AT5G52010	Zinc finger (C2H2 type) family protein	C	+	−	**–**
AT5G62990	Embryo defective 1692 (ubiquitin thiolesterase)	C	+	+	**–**
AT5G64840b)	ATGCN5; *A. thaliana* general control non-repressible 5	C	+	−	**–**

AGI codes of all proteins together with functional annotation from TAIR9 and TargetP prediction are shown. C, chloroplast; M, mitochondrion; S, secretory system; O, other localization. Whether or not a protein was identified with the ATP/PurB and/or Eu^3+^ strategy is depicted by + or −, respectively. When an identified protein exhibits a certain phenotype according to the Chloroplast 2010 database, this is indicated: WP, Whole Plant Morphology; CF, Chlorophyll Fluorescence; SAA, Seed Amino Acid; LFA, Leaf Fatty Acid; LAA, Leaf Amino Acid. Proteins that have been selected for YFP localization study are written in bold. Proteins that have been reported to be localized in the chloroplast during preparation of this publication are labelled by superscript lowercase letters, which are explained at the bottom of the table.

a) Protein is present in the AT_CHLORO database.

b) Protein is chloroplast localized according to the recent PPDB update.

c) Chloroplast-localized according to 71.

According to TargetP 30 out of the 43 putative new chloroplast proteins are predicted to be chloroplast-localized indicating that the majority of putative new proteins are targeted via the canonical import pathway. Interestingly, one protein (AT3G55870) is predicted to enter the secretory pathway. This protein is annotated as subunit of anthranilate synthase, which is an enzyme of the plastidiar-localized shikimate pathway for the synthesis of aromatic amino acids 48. It may be targeted to the chloroplast via the ER, a non-canonical import pathway that has already been described for the carbonic anhydrase CAH1 from *Arabidopsis*, the rice α-amylase AmyI-1 and the rice nucleotide pyrophosphatase/phosphodiesterase NPP1 49–51.

During preparation of this article the new chloroplast protein database AT_CHLORO was launched and also publications confirming the localization of some novel chloroplast proteins were released. In response to these new findings, which nonetheless support the quality of our experimental approach, we reevaluated our putative novel chloroplast proteins (Table 1).

### 3.4 Ligand-binding affinity of identified proteins

We performed affinity chromatography using the ligands ATP, PurB and Eu^3+^. With each affinity ligand we are able to identify a specific subset of proteins (Supporting Information Fig. S2). As expected, the overlap between ATP and PurB was larger (75 proteins) than between ATP and Eu^3+^ (14 proteins) or PurB and Eu^3+^ (39 proteins) reflecting the different nature of the ligand's binding affinities. Nevertheless, even with PurB and ATP several unique proteins could be identified indicating a slightly different mode of action on ATP-binding proteins.

We analyzed all 319 proteins that were identified with the ATP-affinity strategy for the presence of a P-LOOP signature, which is a classical and well-characterized ATP-binding motif 52. While in the whole proteome (TAIR9 release) only 6.3% of all proteins contain a P-LOOP, this motif is enriched to 11.6% within all 319 identified proteins. But it has to be considered that a number of proteins are binding ATP via a completely different motif. As proteins interacting with nucleotides similar to ATP such as FAD, NAD or GTP could have bound to the ATP and PurB columns, we manually investigated all identified proteins for their binding affinities based on their annotated function. In total, 47.7% of the 319 proteins exhibited affinity to ATP or a similar nucleotide. Furthermore, in line with Ito et al. 15, who analyzed the ATP-binding proteome of mitochondria, we identified many classical nucleotide-binding proteins such as HSPs, isoforms of the elongation factor Tu and different dehydrogenases and reductases.

All 273 proteins identified with the Eu^3+^-column were individually analyzed for their ability to bind metal ions based on their annotated function. In total 23% of the proteins are able to bind to Zn, Ca or other metal ions, which is a clear enrichment compared to the average amount of 12% metal-binding (e.g. Zn and Fe) proteins that are present in eukaryotic proteomes according to the analysis of 57 sequenced species using the SCOP (Structural Classification of Proteins) database 53. However, also here it has to be considered that this analysis is only based on available annotations and that therefore the number of genuine metal binding proteins in this data set will most probably be much higher.

### 3.5 Subcellular localization of candidate proteins

As MS detection of proteins in organellar preparations alone is not a convincing proof of localization due to the risk of detecting contaminants, we selected 13 candidates for further experimental investigation by YFP fusion analysis (Table 2; for identified peptides see Supporting Information Table S2).

**Table 2:** The 13 candidate proteins selected for YFP localization

AGI code	Name	Functional annotation (TAIR9)	TargetP	ChloroP	MultiP	Aram.	ATP/PurB	Eu^3+^
AT1G06190	PAP	P-type ATPase, cation-transport	C	C	C	C	−	+
AT1G06510	CUP1	Chloroplast unknown protein 1	C	C	C	C	−	+
AT1G15730	PIF	PRL1-interacting factor L, putative	C	C	C	C	+	−
AT1G19920	APS2	ATP sulfurylase	C	C	C	C	+	−
AT1G22410	DAS	2-Dehydro-3-deoxyphosphoheptonate aldolase	C	C	C	C	+	+
AT1G77670	ATF	Aminotransferase class I and II family protein	M	C	O	O	+	−
AT2G17240	CUP2	Chloroplast unknown protein 1	C	C	C	C	−	+
AT2G25870	HAC	Haloacid dehalogenase-like family protein	M	C	O	M	+	−
AT2G35800	SUC	Substrate carrier family protein	O	O	O	O	−	+
AT3G04650	ORE	FAD-dependent oxidoreductase	C	C	C	C	+	−
AT3G57810	OTL	OTU-like cysteine protease family protein	M	C	C	C	−	+
AT5G16810	PPK	Putative protein kinase	C	O	C	O	+	+
AT5G22620	PGL	Phosphoglycerate mutase family protein	C	C	C	C	+	−

AGI codes of selected proteins, arbitrary name and functional annotation from TAIR9 are shown. YFP indicates the experimentally determined subcellular localization. Results of targeting prediction by TargetP, ChloroP, MultiP and Aramemnon (Aram.) are included as well. C, chloroplast; M, mitochondrion; O, other localization. Whether or not a protein was identified with the ATP/PurB and/or Eu^3+^ strategy is depicted by + or −, respectively.

We chose to analyze the protease OTL, the protein HAC, which belongs to the superfamily of haloacid dehalogenases, the aminotransferase ATF and the two unknown proteins CUP1 and CUP2. Furthermore, we selected the protein PIF, which was shown to interact with the nuclear factor PRL1, the ATP sulfurylase APS2, the 2-dehydro-3-deoxyphosphoheptonate aldolase DAS, the oxidoreductase ORE and the phosphoglycerate mutase PGL. By using relaxed identification criteria we even could identify a putative protein kinase, PPK and an EF-hand containing substrate carrier protein, SUC, which had already been detected in a chloroplast envelope proteomic study 54. Both proteins were also included in our verification experiments. Finally, we added the P-type ATPase PAP to our test set, which had also been identified in a chloroplast proteomics study before, but only with one peptide 11. The subcellular localization of all candidate proteins was analyzed by confocal laser scanning microscopy using C-terminal YFP fusion proteins. In all cases except for SUC full-length coding sequences were used. In the case of SUC only the N-terminal part of the protein was analyzed, but it is known that the N-terminus harboring the cTP is sufficient to mediate chloroplast import 7, 34. For the protein kinase PPK both, the N-terminal part and the full-length protein were analyzed.

**Figure 2:**
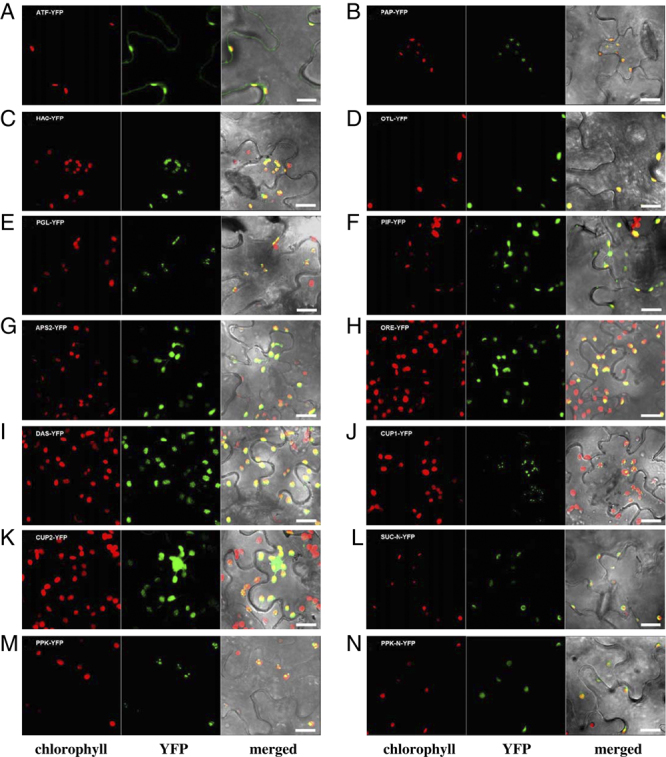
YFP localization of selected candidate proteins. Tobacco leaves infiltrated with constructs in which the gene of interest was fused in front of YFP were analyzed by confocal laser scanning microscopy two days after infiltration. Chlorophyll autofluorescence is shown in the first channel and the YFP signal in the second channel. The third channel is a merged image of the previous two plus transmitted light. N after the name of a protein indicates that only its N-terminus was fused to YFP. Bar = 20 μm.

All of the 13 candidate genes showed chloroplast localization indicated by an overlap of the YFP signal with the autofluorescence signal of chlorophyll (Fig. 2). Interestingly, the proteins PAP, HAC, PGL, DAS, CUP1 and PPK exhibited speckled chloroplast localization, which was similar to the localization pattern of the known chloroplast proteins ferredoxin-NADP^+^ reductase (AT5G66190) and Rubisco activase (AT2G39730) (Supporting Information Fig. S3). In the case of PPK, the speckled pattern could only be observed for the full-length protein but not for the N-terminal portion. This indicated that PPK carries internal information within its protein sequence that is needed to target it to a specific subcompartment within the chloroplast. We suppose that this holds true also for the other proteins with a similar localization pattern.

Furthermore, in both the cases the localization was not exclusively observed in chloroplasts, ATF was detected also in the cytoplasm and CUP2 in the nucleus. This might be an experimental artifact due to overexpression of the proteins using the strong 35S promoter from Cauliflower mosaic virus. But since all other analyzed proteins do not show any background localization to other cellular compartments than the chloroplast, ATF and SUC could also be dually targeted. Furthermore, overexpression of proteins seems to lead to mislocalization rather when multiple copies of the 35S promoter are used. For example the nuclear-localized putative ion channels CASTOR and POLLUX were mistargeted to the chloroplast only when a double 35S promoter was used 55. The most interesting novel chloroplast proteins will be discussed below.

### 3.6 OTL (OTU-like cysteine protease)

OTL belongs to the OTU-like superfamily of predicted cysteine proteases 56. In chloroplasts an unknown cysteine protease activity was shown to be involved in the turnover of Rubisco as well as Rubisco activase and the regulation of the general chloroplast protein composition was effected by overexpression of the cysteine protease inhibitor cystatin in tobacco leaves 57. OTL is now the first identified chloroplast-localized cysteine protease in *Arabidopsis*.

### 3.7 PGL (phosphoglycerate mutase)

Phosphoenolpyruvate, together with erythrose 4-phoshpate, is the precursor of aromatic amino acids synthesized via the shikimate pathway and is therefore a key metabolite in plants. In principle, phosphoenolpyruvate can be formed from 3-phoshoglycerate in two consecutive reaction steps involving a phosphoglycerate mutase, PGL, and an enolase. In *Arabidopsis* the enolase ENO1 was already shown to be localized within the chloroplast 58. In this study we were able to identify the missing chloroplast-localized phosphoglycerate mutase, PGL. During preparation of this article PGL was also identified in another independent chloroplast proteomic study 59.

Interestingly, integrated data analysis of shotgun proteomics and RNA profiling indicated a significant molecular mass bias for the detection of proteins, which are expressed at very low levels 60. This seems to be the case for the plastidiar PGL, thus explaining why its detection by MS had been so difficult. In contrast other metabolic enzymes like transketolase accumulate at much higher levels as related metabolic enzymes 61 or as it would be expected based on their transcript levels 60, even enabling its protein purification from plant tissues 62.

### 3.8 PPK (plastidiar protein kinase)

Protein phosphorylation by protein kinases is a key mechanism to transduce signals within a cell and to regulate processes according to environmental changes. The chloroplast with its numerous metabolic processes is integrated into the cellular signaling network, but so far only a handful chloroplast protein kinases have been identified. Examples are the state transition kinases STN7 and STN8 63, which are involved in photosynthetic acclimation, the plastid transcription kinase CKIIα 64, and the recently described chloroplast sensor kinase CSK 65, which controls transcription of several chloroplast genes. Here, we provide evidence for a novel chloroplast-localized putative protein kinase. Notably, quite a number of different protein kinases are predicted to be localized in chloroplasts but systematic analysis of their localization revealed that most of them are not targeted to chloroplasts in vivo 66. For example, the Ca^2+^-dependent protein kinase CPK3 has a firm prediction for chloroplast targeting, but turned out to be localized to the nucleus and different cellular membranes 67.

### 3.9 SUC (substrate carrier protein)

SUC is a member of the mitochondrial carrier family (MCF), which consists of 58 putative members in *Arabidopsis* 68. Some are known to carry specific substrates not only across the mitochondrial membrane (as the family name might suggest), but also across the chloroplast envelope 69. The identification of SUC in a previous proteomics study of the chloroplast envelope 54 prompted further evaluation of its localization. An N-terminal YFP-fusion protein of SUC clearly localizes to ring-like structures around the chloroplast, hinting at envelope localization. Furthermore, SUC is one of the four predicted MCF proteins to have at least one functional EF-hand. Together, we present here new evidence for a potentially calcium-regulated substrate carrier protein at the chloroplast envelope.

### 3.10 HAC (haloacid dehalogenase)

The haloacid dehalogenase superfamily is a large family of proteins dominated by phosphotransferases. It includes phosphoesterases, ATPases, phosphonatases, dehalogenases and sugar phosphomutases, which act on a remarkably diverse set of substrates and contain a specific form of the Rossmannoid fold 70. Interestingly, eight different haloacid dehalogenase-like proteins, which are evolutionary highly conserved, were also identified in the recent proteomic study by Olinares et al. 43, thus pointing toward an ancient group of regulatory proteins in chloroplast metabolism inherited from their prokaryotic progenitors.

### 3.11 Comparison of the pea EST with the *Arabidopsis* genome database

In order to assess the identification potential of the pea EST database compared to the complete genome database of *Arabidopsis*, we repeated the affinity approach using ATP-Sepharose with chloroplasts isolated from mature *Arabidopsis* plants. The procedure was exactly the same as for pea.

Although the same amount of chloroplasts was used, after gel filtration only 0.82 mg protein could be recovered compared to 1.5 mg with pea, which reflects the well-known fact that during isolation *Arabidopsis* chloroplasts break and lose parts of their stromal content. Remarkably, although less protein was present in the sample, 365 proteins could be identified with *Arabidopsis* in contrast to 234 with pea (Supporting Information Table S3). This is most probably due to the lower sequence coverage of the pea EST database compared to the complete genome database of *Arabidopsis*. Furthermore, the overlap of identified proteins between both organisms accounts for only 160 proteins. This indicates that the usage of pea gave rise to the identification of a different subset of chloroplast proteins, which could be based on two reasons. On the one hand, this is most likely due to species-specific differences in the chloroplast protein content. On the other hand, this probably also reflects differences in the developmental state of the analyzed chloroplasts as seedlings were used for chloroplast isolation from pea, whereas leaves of mature plants were used in the case of *Arabidopsis*.

With the data from the recently published AT_CHLORO database already integrated, out of the 365 *Arabidopsis* proteins 94% were already known to be localized in the chloroplast compared to 86.3% with the pea approach. Strikingly, although approximately 50% more proteins were identified with *Arabidopsis* only nine (2.5%) putative novel chloroplast proteins were found compared to 21 (7.3%) with pea.

## 4 Concluding remarks

At a time where classical top-down organellar proteomic approaches are reaching their detection limits, we have shown that applying a targeted proteomic approach on chloroplasts from the non-model organism pea has the potential to identify novel chloroplast proteins. The use of different affinity ligands could further lead to novel protein identifications and eventually to deeper understanding of chloroplast function.

The comparison of the stromal proteomes of pea and *Arabidopsis* confirmed the expected species- and/or developmental state-specific differences between chloroplasts isolated from mature leaves of *Arabidopsis* and seedlings of pea. Most importantly, the use of the non-model organism pea gave rise to the identification of new chloroplast proteins (e.g. DAS, HAC, ORE) that were not accessible in *Arabidopsis* before. In this context, a further improvement of the targeted approach presented in this study would be the sequencing of the complete pea genome. We predict that usage of a whole genome database for the identification of chloroplast proteins from pea would result in the detection of more (novel) chloroplast proteins, accompanied with a decrease in the contamination rate.

Data on protein identifications associated with this article may be downloaded as Scaffold SFD files from ProteomeCommons.org Tranche using following hashs:

bkNL8osY7uDT6RyhN9K9hbRAKzkUZrH08vjdh7+coXUGYkcPWQbQCTEWmfL/7kQvF7lsXd2L6dm+dExwk1s29tUwTTcAAAAAAAABkw==

tyvgY1UhiPBmFytFIpsUzNsppKE6Gn7oJcPcqP38XFrIMjpYWyVoo6Y8a+chIWvcRjuWDsuHGLuqbyUKXrKgHBirKWgAAAAAAAABkw==

kxF5p9j0zIb1lmQWPgEzHy3HL1iH915WFOI1G/j+B4X6aKA/1FWPhH5hM7+ZZ93B0u59N3dgRu/9Wc6by/DKzvXqaTwAAAAAAAABjQ==

3jYp8J6PZsOY39cTeDsGkT4xx7PP6PMbAQWH37SKb6JK7KcmMkR8ywvq7EafETTBeywuy/R0Aa1Y2AiuCsuBpihn5UwAAAAAAAABtg==

The hashs may be used to prove exactly what files were published as part of this article's data set, and the hash may also be used to check that the data have not changed since publication.

## Abbreviations

AGI: *Arabidopsis* gene identifier
cTP: chloroplast transit peptide
PGL: phosphoglycerate mutase
PPDB: Plant Proteome Database
PPK: putative protein kinase
PurB: purvalanol B
Rubisco: ribulose-1,5-bisphosphate carboxylase/oxygenase
YFP: yellow fluorescent protein

## Supplementary material

Detailed facts of importance to specialist readers are published as ”Supporting Information”. Such documents are peer-reviewed, but not copy-edited or typeset. They are made available as submitted by the authors.
