# Supplementary material for: Mining the soluble chloroplast proteome by affinity chromatography
Source: Proteomics. 2011 Feb 25;11(7):1287–99. doi: 10.1002/pmic.201000495 (PMC3531887; doi:10.1002/pmic.201000495)

PROTEOMICS

Supporting Information for Proteomics

DOI 10.1002/pmic.201000495

Roman G. Bayer, Simon Stael, Edina Csaszar and Markus Teige

Mining the soluble chloroplast proteome by affinity chromatography

© 2011 WILEY-VCH Verlag GmbH & Co. KGaA, Weinheim

FIGURE LEGENDS

Supporting Information Figure S1. Saturation curves of ATP/PurB and Eu^{3+} runs. X-axis shows the number of biological replicates. Y-axis shows identified proteins in percentage of all proteins identified with the respective affinity strategy. The number of uniquely identified proteins with consecutive biological samples was added up.

Supporting Information Figure S2. Comparison of ATP, PurB, and Eu^{3+} affinity runs. Numbers and overlaps of proteins identified by ATP-, PurB-, and Eu^{3+}-affinity chromatography are shown in this Venn diagram.

Supporting Information Fig. S3. YFP localization of known chloroplast proteins. Tobacco leaves expressing ferredoxin-$NADP^{+}$ reductase (A) and Rubisco activase (B) fused in front of YFP were analyzed by confocal laser scanning microscopy. Chlorophyll autofluorescence is shown in the first channel and the YFP signal in the second channel. The third channel is a merged image of the previous two plus transmitted light. Bar = 20 µm.

Bayer et al. suppl. Fig. S1

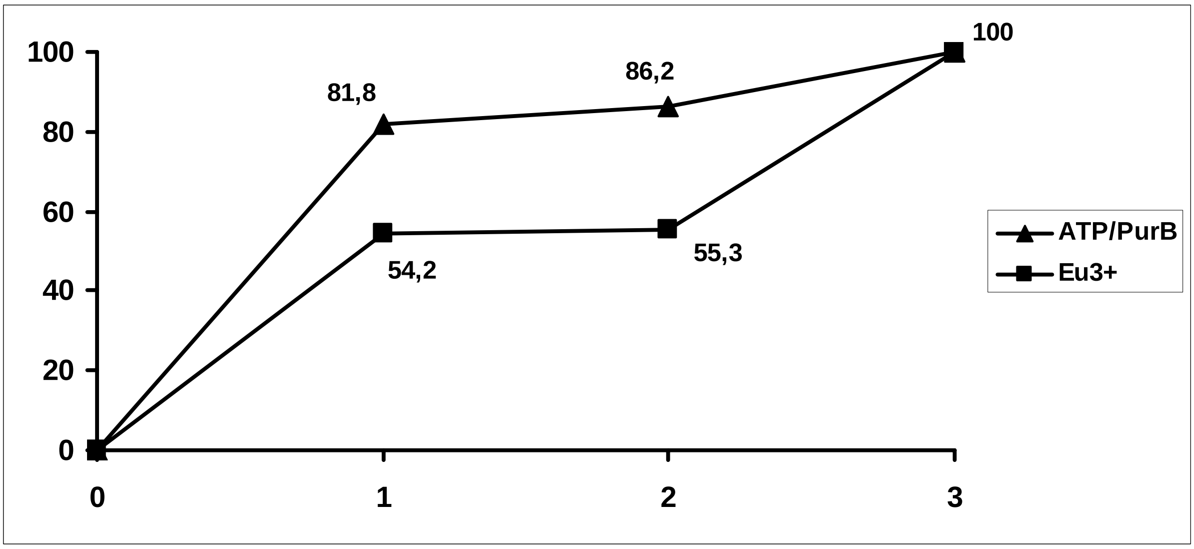

Bayer et al. suppl. Fig. S2

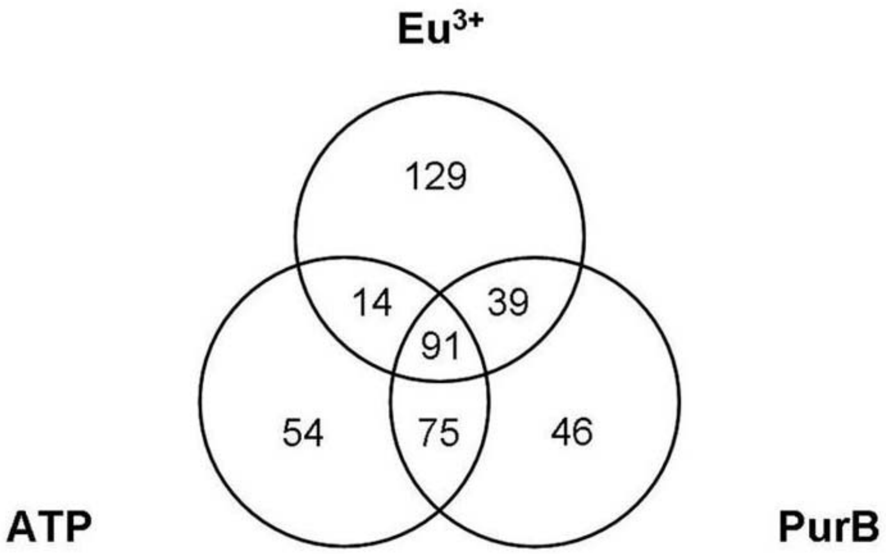

Bayer et al. suppl. Fig. S3

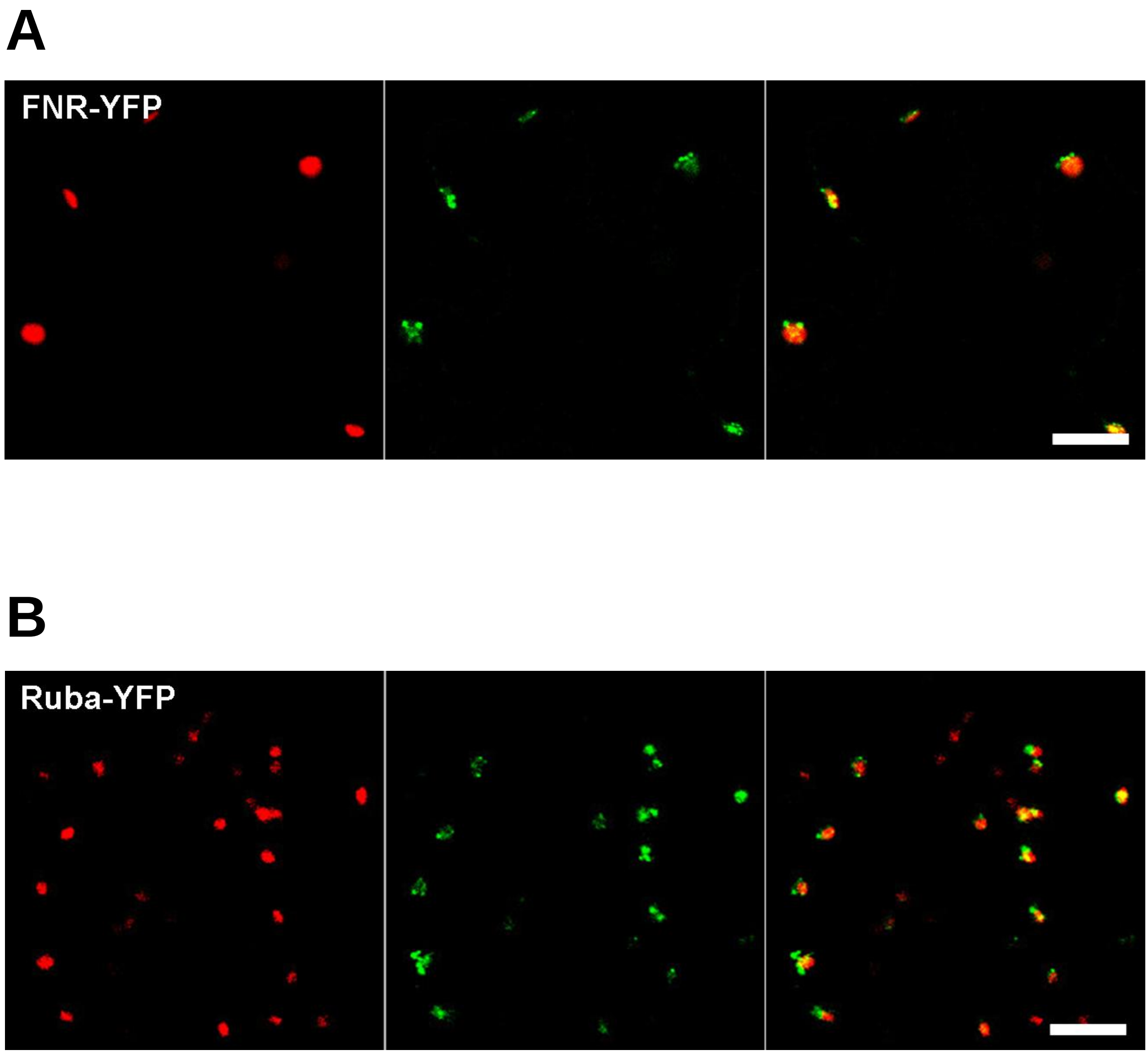

Supplement: Supplementary file 1 [file pmic0011-1287-SD1.pdf]
